# Properties of Taurine Release in Glucose-Free Media in Hippocampal Slices from Developing and Adult Mice

**DOI:** 10.1155/2015/254583

**Published:** 2015-08-05

**Authors:** Simo S. Oja, Pirjo Saransaari

**Affiliations:** Medical School, 33014 University of Tampere, Finland

## Abstract

The release of preloaded [^3^H]taurine from hippocampal slices from developing 7-day-old and young adult 3-month-old mice was studied in a superfusion system in the absence of glucose. These hypoglycemic conditions enhanced the release at both ages, the effect being markedly greater in developing mice. A depolarizing K^+^ concentration accentuated the release, which indicates that it was partially mediated by exocytosis. The anion channel blockers were inhibitory, witnessing the contribution of ion channels. NO-generating agents fomented the release as a sign of the participation of excitatory amino acid receptors. The other second messenger systems were apparently less efficient. The much greater taurine release could be a reason for the well-known greater tolerance of developing nervous tissue to lack of glucose.

## 1. Introduction

Hypoglycemia is a metabolic condition common during development and may lead to severe neurological defects in human infants. However, the effects of hypoglycemia on the developing brain are still incompletely understood [1]. The brain derives most of its energy from the oxidation of glucose, but during development it also has an ability to utilize alternative energy substrates which may offer protection during hypoglycemia [2, 3]. On the other hand, poor reserves of high-energy phosphates and a high metabolic rate may predispose the developing brain to hypoglycemic injury [4, 5]. In the brain the hippocampus is the region most sensitive to lack of oxygen and glucose. Energy deprivation leads to neuronal cell death, caused primarily by excitotoxicity due to excessive glutamate release [6, 7].

Taurine (2-aminoethanesulfonic acid) is present at high concentrations in the brain. During ontogenic development its concentration even exceeds that of the main excitatory transmitter glutamate [8]. It increases membrane chloride conductance, causing hyperpolarization and inhibiting neuronal firing [9, 10]. Taurine also attenuates the excessive neuronal accumulation of Ca^2+^, which predisposes cells to damage [11] and prevents or reduces the glutamate-induced elevation of intracellular Ca^2+^ [12] by inhibiting the glutamate-induced release of Ca^2+^ from the internal pools [13] and the glutamate-induced Ca^2+^ influx through L-, P/Q-, and N-types of voltage-gated Ca^2+^ channels [14]. Taurine thus regulates cytoplasmic and mitochondrial calcium homeostasis [15] and in this manner protects neural cells against the toxicity of excitatory amino acids in the hippocampus [16]. Cell-damaging conditions, including hypoglycemia, increase the release of taurine [17, 18] together with that of excitatory amino acid neurotransmitters. Our primary assumption is that taurine release in the absence of an adequate supply of glucose could protect neural cells from injury. In the present study we therefore examined the general properties of taurine release in the hippocampus in hypoglycemia and how the release is affected by ion channels, second messenger systems, and adenosine receptors.

## 2. Materials and Methods

### 2.1. Materials

Developing (7-day-old) and young adult (3-month-old) NMRI mice of both sexes were used in the experiments. All efforts were made to minimize both the suffering and the number of the animals used. The experiments conformed to the European Community Directive (86/609/EEC) for ethical use of experimental animals and were approved by the Committee of Tampere University for animal experiments. [^3^H]Taurine (specific radioactivity 1.15 PBq/mol) was obtained from Amersham International, Bristol, UK. The various effectors were purchased from the Tocris Bioscience (Bristol, UK) or Sigma Aldrich (St. Louis, MO).

### 2.2. Release Experiments

Coronal slices 0.4 mm thick weighing 15–20 mg were manually prepared from the mouse hippocampus with a tissue slicer of Stadie-Riggs type. The slices were immediately immersed in 5 mL of oxygenated medium and incubated with 0.01 mM [^3^H]taurine (50 MBq/L) at 37°C for 30 min under agitation. The standard Krebs-Ringer-Hepes medium contained (in mmol/L) NaCl 127, KCl 5, CaCl_2_ 0.8, MgSO_4_ 1.3, Na_2_HPO_4_ 1.3, N-2-hydroxyethylpiperazine-N′-2-ethanesulphonic acid (Hepes) 15, NaOH 11, and D-glucose 10 (pH 7.4). The slices were then transferred into 0.25 mL cups and superfused with the above medium at a rate of 0.25 mL/min for 50 min in a system in which freely floating shaken slices were kept under a continuous flow of oxygen in order to preserve their viability [19]. Hypoglycemic conditions were induced by omitting glucose from the superfusion media. Potassium stimulation was applied from 30 to 50 min with 50 mM K^+^. In our experimental setup this K^+^ concentration has yielded the best and most reproducible responses in GABA and taurine release [19]. This high K^+^ concentration may cause release not only from neurons but from glial cells as well [20]. However, taurine release is typically slow at onset and prolonged and high K^+^ concentrations above those prevailing in vivo should be used in in vitro experiments [21]. The different effectors were added to the medium at the onset of superfusions, as explained in the table legends. The superfusion medium was pooled during the first 20 min, whereafter 2 min fractions (0.5 mL) were collected directly into small scintillation vials with a fraction collector. After superfusion the slices were weighed, homogenized in ice-cold 5% (w/v) trichloroacetic acid solution, and centrifuged, and the clear supernatants were used for scintillation counting. The effluent samples were subjected to the same analyses.

### 2.3. Estimation of Efflux Rate Constants

Desaturation curves of labeled taurine from the slices were plotted as a function of time on the basis of the radioactivities remaining in the slices after superfusion and recovered in the collected superfusate fractions [19]. During superfusion the release of labeled taurine originates initially from the extracellular spaces in slices. This source is gradually exhausted during the first 20 min and the release subsequently occurs from the intracellular pools. The efflux rate constants of taurine for the time intervals of 20 to 30 min (*k*_1_, initial release phase) and 34–50 min (*k*_2_, later release phase) were computed as negative slopes for the regression lines of the logarithm of radioactivity remaining in the slices versus superfusion time. There were no differences between the results from male and female mice and the results from both sexes were therefore combined.

### 2.4. Statistical Analysis

The significance of the results was analyzed with two-way analysis of variance (ANOVA) using SPSS statistics, version 17.0, computer program. The analyses were done by grouping the results according to the nature of the effectors studied, potassium stimulation, chloride channel blockers, NO-generating agents, adenosine agonists, and second messengers. When significant effects were detected, the post hoc Bonferroni test was applied to bring out the differences between the sample means. They were considered significant when the calculated *p* values were less than 0.05 or 0.01.

## 3. Results

Hypoglycemia significantly enhanced taurine release in both age groups. In adult 3-month-old mice the fractional release rate constant in the presence of glucose in medium was for the superfusion period of 34–50 min (1.54 ± 0.04) × 10^−3^ (*n* = 18) and in the absence of glucose (1.93 ± 0.18) × 10^−3^, *n* = 11, significantly different at a level of* p* = 0.042. The hypoglycemia effect was more marked in 7-day-old mice, the corresponding constants being (0.38 ± 0.20) × 10^−3^, *n* = 11, and (1.20 ± 0.12) × 10^−3^, *n* = 11,* p* = 0.000, respectively. Stimulation by 50 mM K^+^ enhanced the release in the absence of glucose (df = 3, *F* = 23.007, *p* = 0.000). The enhancements were 60 per cent and 63 per cent in adult (*p* = 0.000) and developing (*p* = 0.001) mice, respectively (see Table 1).

The anion channel blockers generally inhibited the release (df = 7, *F* = 13.562, *p* = 0.000). Of them, DIDS significantly inhibited the K^+^-stimulated taurine release in both adult (*p* = 0.013) and developing (*p* = 0.044) mice, being in adult mice also effective on the unstimulated release (*p* = 0.047) (Table 1). SITS, the blocker of chloride transport, was even more effective in all experimental situations (in adult mice, unstimulated release *p* = 0.11, stimulated release, *p* = 0.035, and in developing mice the corresponding data *p* = 0.016 and *p* = 0.017). Another transport inhibitor 9-AC was not effective. All nitric oxide generators, SNAP, SNP, and hydroxylamine, were strong stimulators in taurine release in both adult (df = 7, *F* = 32.190, *p* = 0.000) and developing (df = 7, *F* = 13.732, *p* = 0.000) mice and in both the unstimulated and K^+^-stimulated release (Table 2). On the other hand, all adenosine receptor agonists tested, CHA, R-PIA and CGS 21680, and riluzole, which inhibits glutamate release and GABA uptake, were without any effects (Table 3).

Of compounds involved in the second messenger systems, genistein, with its main known activity as a tyrosine kinase inhibitor, was not effective during the period of 34–50 (Table 4), whereas quinacrine, a nonselective inhibitor of both monoamine oxidases A and B, inhibited the unstimulated and K^+^-stimulated release in both age groups at all stages of superfusion (df = 6, *F* = 23.648, *p* = 0.000). The protein kinase C activator PMA only inhibited the unstimulated release in adult mice (*p* = 0.036), whereas the protein kinase inhibitor chelerythrine was not effective. The nonspecific inhibitor of cAMP and cGMP phosphodiesterases and the nonselective adenosine receptor antagonist IBMX was not effective during the period of 34–50 min. The selective inhibitor of cGMP-insensitive phosphodiesterase, type IV, RO 20-1724 enhanced both the unstimulated (*p* = 0.008) and stimulated (*p* = 0.005) release in adult mice and also slightly the stimulated release (*p* = 0.038) in developing mice (Table 4). The selective inhibitor of cGMP-specific phosphodiesterases V and VI (PDE5/6) and the agonist at the G protein-coupled receptor 35 zaprinast significantly (*p* = 0.035) reduced the unstimulated release in 7-day-old mice, whereas ODQ, the potent and selective inhibitor of NO-sensitive guanylyl cyclase, was without effect. Alloxan, the unspecific inhibitor of adenylyl cyclases, was likewise not effective during the period of 34–50 min.

## 4. Discussion

The present results show taurine release to be enhanced in hypoxia at both ages studied, the effect being more pronounced in the developing hippocampus. The release is largely mediated by anion channels, since the anion channel inhibitors markedly reduced it. The nitric acid generators strongly stimulated the release. The effects of the second messenger system were more variable. These actions were also in many cases different in adult and developing mice.

The enhancement could be due to several mechanisms, including Ca^2+^-dependent exocytosis, Ca^2+^-independent release via reversal of carrier-mediated uptake, indiscriminate opening of ion channels which allow the passage of taurine molecules, or leakage through damaged plasma membranes. Depolarization by K^+^-stimulation was now able to further potentiate taurine release at both ages under hypoglycemia. This may signify release via exocytosis, which is also preserved in hippocampal slices when they are exposed to even more drastic cell-damaging conditions such as ischemia (the absence of glucose and oxygen atmosphere) [17, 18]. In developing mice taurine release in the absence of glucose is approximately the same as in the presence of nitrogen atmosphere without glucose, whereas in adults these ischemic conditions have had even more impact [18]. Both neurons and glial cells have been shown to contain taurine [22], and thus only a part of the released taurine may originate from neurons. K^+^ stimulation of taurine release has also been shown to be associated with cell swelling [23]. Indeed, the volume-regulated anion channels may contribute, together with the reversed function of astrocytic glutamate transporters, up to 80 per cent of the total release of taurine and excitatory amino acids in global cerebral ischemia in rats [24].

Intracellular swelling activates stretch-sensitive (volume-regulated) ion channels and is accompanied by the release of both inorganic and organic osmolytes, including taurine [25]. For instance, the volume-regulated anion channels have been shown to be the predominant contributors to the release of excitatory amino acids in the ischemic cortical penumbra [26]. The swelling-induced increase in taurine release is a diffusional process without any carrier involvement [27].

In both the developing and adult hippocampus the presynaptically acting ionotropic glutamate agonists significantly increase the release of taurine in a receptor-mediated manner [28, 29]. For instance, of the ionotropic receptors, NMDA receptor activation in particular has been assumed to play a central role in hypoglycemia-induced glutamate release [30]. Activation of NMDA receptors allows Ca^2+^ to enter the cells. NO synthase is a Ca^2+^-dependent enzyme [31], being activated in the presence of Ca^2+^ and then producing NO. NO stimulates soluble guanylate cyclase and in this manner foments the production of 3′,5′-cyclic guanosine monophosphate [32], which enhances taurine release [33]. The present activatory effect of the NO generators studied is in concert with this sequence of events. NO also regulates neuronal activity by activating calcium-dependent potassium channels [34, 35]. NO can be the endogenous opener of leak K^+^ channels in the hippocampus as shown in basal forebrain cholinergic neurons [36]. The increase in extracellular K^+^ enhances taurine release. The NO donors did not markedly foment the K^+^-evoked release now. The effect was apparently already maximal at the K^+^ concentration used in this study.

NO produced by an exogenous NO donor has been shown to block both tetrodotoxin-sensitive and tetrodotoxin-resistant Na^+^ currents in baroreceptor neurons [37] and NO to block different Na^+^ channels in baroreceptor neurons [38]. In contrast, NO donors SNP and SNAP have significantly increased the mean persistent current in excised inside-out patches from cultured hippocampal neurons [39]. Such results suggest that, depending on cell type, NO may modulate Na^+^ currents differently. Taurine efflux has been shown to be dependent on the presence of both Na^+^ and Cl^−^ ions [40]. The reduction of release by the Cl^−^ channel antagonists SITS and DIDS indicates that the release may also occur through anion channels, similar to the volume-sensitive taurine release in astrocytes and neurons [41, 42]. The present attenuating effect of quinacrine on the release is furthermore in line with this assumption since quinacrine has been shown to inhibit the hypotonically induced whole cell Cl^−^ currents in human submandibular gland (HSG) cells [43].

The various second messengers exerted less pronounced effects on taurine release. In keeping with this the effects of different metabotropic glutamate receptor agonists and antagonists have also not been particularly effective in taurine release in the hippocampus [44]. The presynaptic adenosine receptors, particularly of the A_1_ class, are known to regulate neurotransmitter release [45]. The A_1_ receptors have also enhanced taurine release in the adult hippocampus, but only when it was subjected to K^+^ stimulation in ischemia [46]. In the present study the nonspecific antagonist of adenosine receptors likewise exhibited only minor effects.

## 5. Conclusions

The release of taurine in the hypoglycemic hippocampus of both young and adult mice appears to be a complex process mediated by several different mechanisms, such as exocytosis, carrier reversal, diffusion via ion channels, and probably leakage through partially damaged cell membranes. Hypoglycemia also evokes the release of excitatory amino acids [6, 18]. Glutamate release overactivates its receptors and functions as an excitotoxic agent. There is a rationale here in that the activation of excitatory amino acid receptors causes a concomitant increase in taurine release. Taurine is inhibitory in nature and counteracts this excessive excitation. In the developing brain taurine release is much larger in magnitude than in the adult brain, which could be a reason for the well-known greater tolerance of developing nervous tissue to lack of glucose.

## Figures and Tables

**Table 1:** Effects of ion channel inhibitors on taurine release from hippocampal slices from 3-month-old and 7-day-old mice in hypoglycemia.

Effectors	Efflux rate constants (×10^−3^ min^−1^) ± SEM							
	3-month-old				7-day-old			
	*k*_1_		*k*_2_		*k*_1_		*k*_2_	
Basal (control)	2.36 ± 0.11	(20)	1.93 ± 0.18	(11)	1.34 ± 0.08	(31)	1.20 ± 0.11	(11)
+50 mM K^+^ (control)			2.92 ± 0.21	(10)			1.90 ± 0.10	(12)

DIDS 0.5 mM	1.34 ± 0.15^*∗∗*^	(6)	1.31 ± 0.10^*∗*^	(4)	1.12 ± 0.14	(8)	1.16 ± 0.13	(4)
+50 mM K^+^			1.81 ± 0.12^*∗∗*^	(4)			1.45 ± 0.06^*∗*^	(4)

SITS 2.0 mM	1.04 ± 0.07^*∗∗*^	(8)	0.93 ± 0.06^*∗∗*^	(4)	0.80 ± 0.09^*∗∗*^	(6)	0.62 ± 0.06^*∗∗*^	(4)
+50 mM K^+^			1.50 ± 0.21^*∗∗*^	(4)			0.90 ± 0.12^*∗∗*^	(4)

9-AC 0.2 mM	2.67 ± 0.17	(5)	2.74 ± 0.49	(4)	1.82 ± 0.23	(8)	1.07 ± 0.09	(4)
+50 mM K^+^			3.53 ± 0.32	(4)			1.84 ± 0.10	(4)

The drugs were added at the beginning of superfusion and 50 mM K^+^ at 30 min. The results show the efflux rate constants ± SEM (×10^−3^ min^−1^) for the time intervals of 20–30 min (*k*_1_) and 34–50 min without the excess of K^+^ or in the presence of 50 mM K^+^ (*k*_2_) with the number of independent experiments in parenthesis. Abbreviations: DIDS, diisothiocyanostilbene-2′2-disulphonate; SITS, 4-acetamido-4′-isothiocyanostilbene-2′2-disulphonate; 9-AC, 9-anthracenecarboxylic acid. Significance of differences from the corresponding controls: ^*∗*^ *p* < 0.05, ^*∗∗*^ *p* < 0.01.

**Table 2:** Effects of nitric oxide generators on taurine release from hippocampal slices from 3-month-old and 7-day-old mice in hypoglycemia.

Effectors	Efflux rate constants (×10^−3^ min^−1^) ± SEM							
	3-month-old				7-day-old			
	*k*_1_		*k*_2_		*k*_1_		*k*_2_	
Basal (control)	2.36 ± 0.11	(20)	1.93 ± 0.18	(11)	1.34 ± 0.08	(31)	1.20 ± 0.11	(11)
+50 mM K^+^ (control)			2.92 ± 0.21	(10)			1.90 ± 0.10	(12)

SNAP 1.0 mM	4.31 ± 0.22^*∗∗*^	(7)	5.44 ± 0.14^*∗∗*^	(4)	2.84 ± 0.11^*∗∗*^	(8)	2.17 ± 0.07^*∗∗*^	(4)
+50 mM K^+^			5.35 ± 0.15^*∗∗*^	(4)			2.34 ± 0.08^*∗∗*^	(4)

SNP 1.0 mM	3.17 ± 0.13^*∗∗*^	(15)	4.23 ± 0.38^*∗∗*^	(8)	1.80 ± 0.21^*∗*^	(7)	1.65 ± 0.13^*∗*^	(4)
+50 mM K^+^			4.41 ± 0.77^*∗∗*^	(4)			1.98 ± 0.10^*∗∗*^	(4)

Hydroxylamine 5.0 mM	5.16 ± 0.17^*∗∗*^	(7)	6.04 ± 0.76^*∗∗*^	(7)	3.82 ± 0.33^*∗∗*^	(8)	3.44 ± 0.16^*∗∗*^	(4)
+50 mM K^+^			7.03 ± 0.28^*∗∗*^	(4)			3.84 ± 0.10^*∗∗*^	(4)

The effectors were added at the beginning of superfusion and 50 mM K^+^ at 30 min. The results show the efflux rate constants ± SEM (×10^−3^ min^−1^) for the time intervals of 20–30 min (*k*_1_) and 34–50 min without the excess of K^+^ or in the presence of 50 mM K^+^ (*k*_2_) with the number of independent experiments in parenthesis. SNAP: S-nitroso-N-acetylpenicillamine; SNP: sodium nitroprusside. Significance of differences from the corresponding controls: ^*∗*^ *p* < 0.05, ^*∗∗*^ *p* < 0.01.

**Table 3:** Effects of adenosine agonists and riluzole on taurine release from hippocampal slices from 3-month-old and 7-day-old mice in hypoglycemia.

Effectors	Efflux rate constants (×10^−3^ min^−1^) ± SEM							
	3-month-old				7-day-old			
	*k*_1_		*k*_2_		*k*_1_		*k*_2_	
Basal (control)	2.36 ± 0.11	(20)	1.93 ± 0.18	(11)	1.34 ± 0.08	(31)	1.20 ± 0.11	(11)
+50 mM K^+^ (control)			2.92 ± 0.21	(10)			1.90 ± 0.10	(12)

CHA 0.5 mM	2.98 ± 0.12	(6)	1.85 ± 0.30	(6)	1.48 ± 0.07	(10)	1.11 ± 0.06	(7)
+50 mM K^+^			2.42 ± 0.27	(4)			2.00 ± 0.06	(4)

R-PIA 0.1 mM	2.46 ± 0.17	(6)	1.92 ± 0.21	(6)	1.97 ± 0.19	(6)	1.41 ± 0.12	(4)
+50 mM K^+^			3.63 ± 0.51	(4)			2.12 ± 0.11	(4)

CGS 21680 10.0 mM	2.59 ± 0.22	(4)	2.20 ± 0.20	(4)	1.75 ± 0.16	(6)	1.38 ± 0.20	(4)
+50 mM K^+^			3.44 ± 0.32	(4)			2.28 ± 0.11	(4)

Riluzole 0.1 mM	2.47 ± 0.17	(7)	1.86 ± 0.27	(4)	1.86 ± 0.15^*∗*^	(6)	1.38 ± 0.10	(4)
+50 mM K^+^			2.50 ± 0.35	(4)			2.23 ± 0.15	(4)

The agonists were added at the beginning of superfusion and 50 mM K^+^ at 30 min. The results show the efflux rate constants ± SEM (×10^−3^ min^−1^) for the time intervals of 20–30 min (*k*_1_) and 34–50 min without the excess of K^+^ or in the presence of 50 mM K^+^ (*k*_2_) with the number of independent experiments in parenthesis. CHA: N^6^-cyclohexyladenosine; R-PIA: (R(−)N^6^-(2-phenylisopropyl)adenosine; CGS 21680: 4-[2-[[6-amino-9-(*N*-ethyl-*β*-D-ribofuranuronamidosyl)-9*H*-purin-2-yl]amino]ethyl]benzenepropanoic acid hydrochloride. Significant difference from the control: ^*∗*^ *p* < 0.05.

**Table 4:** Effects of compounds involved in the second messenger systems on taurine release from mouse hippocampal slices in hypoxia.

Concentration (mM)	Efflux rate constants (×10^−3^ min^−1^) ± SEM							
	3-month-old				7-day-old			
	*k*_1_		*k*_2_		*k*_1_		*k*_2_	
Basal (control)	2.36 ± 0.11	(20)	1.93 ± 0.18	(11)	1.34 ± 0.08	(31)	1.20 ± 0.11	(11)
+50 mM K^+^ (control)			2.92 ± 0.21	(10)			1.90 ± 0.10	(12)

Genistein 0.001	2.59 ± 0.13	(7)	2.06 ± 0.18	(7)	1.92 ± 0.16^*∗*^	(7)	1.44 ± 0.16	(4)
+50 mM K^+^			3.07 ± 0.25	(4)			2.27 ± 0.06	(4)

Quinacrine 0.01	1.20 ± 0.08^*∗∗*^	(7)	0.83 ± 0.05^*∗∗*^	(4)	0.75 ± 0.07^*∗∗*^	(8)	0.61 ± 0.06^*∗∗*^	(4)
+50 mM K^+^			1.12 ± 0.13^*∗∗*^	(4)			0.79 ± 0.05^*∗∗*^	(4)

PMA 0.00001	2.31 ± 0.20	(8)	1.77 ± 0.11	(4)	1.80 ± 0.15^*∗*^	(8)	1.33 ± 0.17	(4)
+50 mM K^+^			1.95 ± 0.17^*∗*^	(4)			1.85 ± 0.03	(4)

Chelerythrine 0.001	2.49 ± 0.17	(7)	2.07 ± 0.14	(4)	1.73 ± 0.18	(8)	1.53 ± 0.04	(4)
+50 mM K^+^			3.40 ± 0.51	(4)			2.01 ± 0.09	(4)

IBMX 1.0	1.85 ± 0.16	(8)	2.51 ± 0.29	(4)	1.99 ± 0.24	(6)	1.33 ± 0.11	(4)
+50 mM K^+^			2.70 ± 0.13	(4)			1.85 ± 0.03	(4)

RO 20-1724 0.2	3.46 ± 0.12^*∗∗*^	(8)	3.43 ± 0.15^*∗∗*^	(4)	1.96 ± 0.26	(7)	1.27 ± 0.17	(4)
+50 mM K^+^			4.56 ± 0.30^*∗∗*^	(4)			2.65 ± 0.33^*∗*^	(4)

Zaprinast 0.1	2.31 ± 0.15	(8)	2.00 ± 0.20	(8)	1.00 ± 0.13	(6)	0.66 ± 0.12^*∗*^	(4)
+50 mM K^+^			2.53 ± 0.06	(4)			2.04 ± 0.23	(4)

ODQ 0.01	2.51 ± 0.18	(7)	2.01 ± 0.13	(4)	1.50 ± 0.17	(8)	1.19 ± 0.08	(8)
+50 mM K^+^			2.50 ± 0.25	(4)			2.04 ± 0.06	(4)

Alloxan 5.0	2.81 ± 0.31	(7)	2.19 ± 0.06	(4)	1.70 ± 0.17	(6)	1.58 ± 0.25	(4)
+50 mM K^+^			3.13 ± 0.10	(4)			1.86 ± 0.07	(4)

The agonists were added at the beginning of the superfusion and 50 mM K^+^ at 30 min. The results show the efflux rate constants ± SEM (×10^−3^ min^−1^) for the time intervals of 20–30 min (*k*_1_) and for 34–50 min without the excess of K^+^ or in the presence of 50 mM K^+^ (*k*_2_) with the number of independent experiments in parenthesis. PMA: 4*β*-phorbol 12-myristate 13-acetate; IBMX: 3-isobutyl-1-methylxanthine; ODQ: 1H-[1,2,4]oxadiazolo[4,3]quinoxalin-1-one; RO 20-1724: 4-(3-butoxy-4-methoxyphenyl)-2-imidazolidone. Significance of differences from the corresponding controls: ^*∗*^ *p* < 0.05, ^*∗∗*^ *p* < 0.01.

## Abbreviations

9-AC: 9-Antracenecarboxylate
AIDA: (RS)-1-Aminoindan-1,5-dicarboxylate
AMPA: 2-Amino-3-hydroxy-5-methyl-4-isoxazolepropionate
2R,4R-APDC: (2R,4R)-4-Aminopyrrolidine-2,4-dicarboxylate
cAMP: Cyclic adenosine monophosphate
ATPase: Adenosine triphosphatase
cGMP: Cyclic guanosine monophosphate
CGS 21680: 4-[2-[[6-Amino-9-(*N*-ethyl-*β*-D-ribofuranuronamidosyl)-9*H*-purin-2-yl]amino]ethyl]benzenepropanoic acid hydrochloride
CHA: N^6^-Cyclohexyladenosine
DHPG: (S)-3,5-Dihydroxyphenylglycine
DIDS: Diisothiocyanostilbene-2,2′-disulphonate
DMPX: 3,7-Dimethyl-1-propargylxanthine
EDTA: Ethylenediaminetetraacetate
EGLU: (2S)-2-Cyclopropyl-4-phosphonophenylglycine
GABA: *γ*-Aminobutyrate
GAT: GABA transporter
HA: Hydroxylamine
Hepes: N-2-Hydroxyethylpiperazine-N′-2-ethanesulphonic acid
IBMX: 3-Isobutyl-1-methylxanthine
L-AP4: L(+)-2-Amino-4-phosphonobutyrate
L-NAME: N^6^-Nitro-L-argininemethylester
L-SOP: O-Phospho-L-serine
MK-801 (dizocilpine): (5S,10R)-(+)-Methyl-10,11-dihydro-5*H*-dibenzo(a,d)cyclohepten-5,10-amine
NBQX: 2,3-Dioxo-6-nitro-1,2,3,4-tetrahydrobenzo[f]quinoxaline-7-sulfonamide
NMDA: N-Methyl-D-aspartate
ODQ: 1H-(1,2,4)Oxadiazolo(4,3-a)quinoxalin-1-one
PDE: Phosphodiesterase
PMA: 4*β*-Phorbol 12-myristate 13-acetate
PKC: Protein kinase C
RO 20-1724: 4-(3-Butoxy-4-methoxyphenyl)-2-imidazolidone
R-PIA: R(−)N^6^-(2-Phenylisopropyl)adenosine
SITS: 4-Acetamido-4′-isothiocyanostilbene-2,2′-disulphonate
SNAP: S-Nitroso-N-acetylpenicillamine
SNP: Sodium nitroprusside.
